# Socioeconomic variation in colon cancer tumour factors associated with poorer prognosis

**DOI:** 10.1038/sj.bjc.6601192

**Published:** 2003-08-26

**Authors:** G Lyratzopoulos, C R West, E M I Williams

**Affiliations:** 1 Evidence for Population Health Unit, University of Manchester, UK; 2 Department of Public Health, University of Liverpool, UK

## Abstract

Analysis of population-based registry data (*n*=7393) showed that more deprived colon cancer patients had lower risk of the mucin-producing adenocarcinoma subtype, proximal subsite (to the descending colon), and no greater risk of high-grade tumours. Tumour factors therefore appear unlikely to account for socioeconomic gradients in survival.

Colorectal cancer survival is greater in more affluent UK patients (Kogevinas *et al*, 1991; Schrijvers *et al*, 1995; Pollock and Vickers, 1997; Coleman *et al*, 1999; Wrigley *et al*, 2003). For patients diagnosed in the early 1990s, the 5-year relative survival deficit between most- and least-deprived patients was 4% (Coleman *et al*, 1999). Patient, healthcare or tumour factors may be responsible (Kogevinas and Porta, 1997). The role of socioeconomic (SE) differences in tumour factors as a potential explanation for differences in survival is uncertain. A plausible hypothesis is that more deprived patients are more likely to have tumours with more aggressive characteristics.

Known tumour factors associated with a poor outcome in colon cancer are mucin-producing adenocarcinoma (MPA) sub-type, proximal subsite and high tumour grade (poorly differentiated tumours) (Mayberry *et al*, 1995; Chen *et al*, 1997; Niv, 2000). Of the above factors, colon cancer subsite is the one best studied. Variations have been observed in subsite-specific incidence between sexes, and ethnic and SE groups (Young and Wolf, 1988; Faivre J *et al*, 1989; Jass, 1991; Levi *et al*, 1991; Kravdal *et al*, 1993, Mayberry *et al*, 1995; Chen *et al*, 1997). There are also differential time trends in the incidence of proximal and distal sub-sites (Snyder *et al*, 1977; Beart *et al*, 1983; Vobecky *et al*, 1984; Jass, 1991; Kee *et al*, 1992). The above indicate the existence of different risk factors for various colon cancer subsites, not only genetic but also environmental. A study was therefore conducted to examine whether more deprived colon cancer patients are more likely to have tumours of proximal subsite, and also, MPA subtype and high grade.

## METHODS

The Merseyside and Cheshire Cancer Registry database incorporates information directly extracted from pathology reports and clinical case notes. Data were obtained for all colon cancer cases during 1989–1996. The overall registry data quality is high and compares favourably with other registries (Seddon and Williams, 1997). Cases were excluded if they were <50 years old, or had multiple colon cancer registrations (synchronous or metachronous), due to high probability of familial or hereditary colon cancer in such patients. Carcinoid and other neuroendocrine tumours were also excluded.

Socioeconomic status is commonly measured directly (i.e. by measuring individuals' income, occupation or education) or indirectly by using area-based measures (i.e. based on the predominant characteristics of the population of a small area) (Liberatos *et al*, 1988). When using area-based measures, there is a potential for misclassification error in the ascertainment of SE status. In this study, Carstair's deprivation index, a census-based ecological measure of SE status, was used as an indicator of SES (Carstairs and Morris, 1992), in common with previous UK research in this field (Pollock *et al*, 1997; Coleman *et al*, 1999; Wrigley *et al*, 2003) and due to lack of individual-level information. Quintile groups were defined, calculated for England and Wales (1991 census). The first group is the least deprived (taken as the reference group) and the fifth group the most deprived.

Age group and histopathological subtype were categorised as shown in Table 1

**Table 1:** Basic characteristics of study population

	**No**	**%**
**Variable (% completeness)**	**6932**	**100**
*Sex (100)*		
Male	3302	47.6
Female	3630	52.4
Unknown	0	0
*Age group (100) (years)*		
50–64	1549	22.3
65–74	2290	33
75 and over	3093	44.6
Unknown	0	0
Deprivation groups (98.5)		
Affluent	1174	16.9
Group 2	902	13
Group 3	431	6.2
Group 4	1648	23.8
Deprived	2672	38.5
Unknown	105	1.5
Histopathol. subtype (77.7)		
Mucin-producing types	430	6.2
Non-mucinous types	4757	71.5
Unknown	1545	22.3
*Subsite (72.5)*		
Ascending	1981	28.6
Transverse	404	5.8
Descending	518	7.5
Sigmoid	2023	29.2
Overlapping	101	1.5
Unknown	1905	27.5
*Histological grade (42.5)*		
Grade 1	469	6.8
Grade 2	2118	30.6
Grade 3	365	5.3
Unknown	3981	57.5

. Subsite information was available to the ICD-O three-digit code level. Subsites were aggregated into ‘distal’ (descending and sigmoid), ‘proximal’ (all other subsites) and ‘unknown/overlapping’, in a way similar to previous research (Jass, 1991; Kee *et al*, 1992). Tumour grade was categorised as ‘poor’ (grade 3), ‘not poor’ (grades 1 and 2) and ‘unknown’ (grades 1, 2 and 3, implying good, moderate and poor differentiation, respectively).

### Statistical analysis

‘Unknown’ status for tumour subsite, type, grade and diagnosis based on histology was tested for association with deprivation status using the χ^2^ test for trend. Binary logistic regression models were used to examine the likelihood of MPA tumour subtype (model 1), proximal subsite (model 2) and poor grade (model 3), respectively, by deprivation group, adjusting for sex and age group. Subsite was also adjusted for in model 1 and subtype in models 2 and 3.

Cases for which the independent variable was unknown were excluded. Cases for which dependent variables were unknown were included in all models, treated as a separate category. The test for trend in the effect of SES was based on regression analysis with Carstairs' score entered as a continuous variable (range from −5 to 15). The Odds ratio (ORs) derived by the test for trend indicates the effect of one unit change in deprivation status on the probability of the outcome under examination.

## RESULTS

There were 7393 cases, of which 461(6.3%) were excluded (Table 1). Most exclusions (4.8%) were in those <50 years old, 0.9% due to pathology other than carcinoma and 0.6% due to multiple colon cancer registrations. In 5187 (77.7%) cases, the diagnosis was based on histology. Completeness for other variables is shown in Table 1. There was no association between deprivation and incomplete ascertainment status, for either subsite, type, grade and diagnosis not based on histology (χ^2^ for trend *P*-values: 0.54, 0.67, 0.53 and 0.67, respectively).

Having adjusted for sex, age group and subsite, MPA subtype was less likely in more deprived patients (Table 2–model 1

**Table 2:** Probability of MPA tumour type, proximal subsite and poor grade by deprivation group (adjusted for sex, age group and relevant tumour factors)

	**MPA subtype (model 1, *n*=6827)**	**Proximal subsite (model 2, *n*=4853)**	**Poor grade (model 3, *n*=2536)**
Deprivation			
Affluent	*1.0*	*1.0*	*1.0*
Group 2	0.87 (0.62-1.22)	1.15 (0.93 - 1.41)	0.63 (0.39-1.03)
Group 3	0.77 (0.69-1.21)	1.01 (0.77 - 1.31)	0.77 (0.42-1.41)
Group 4	0.86 (0.65-1.17)	1.07 (0.89 - 1.27)	1.14 (0.79-1.65)
Deprived	0.63 (0.45-0.84)***	0.93 (0.78 - 1.09)	0.88 (0.61-1.26)

*Change/unit*†	0.96 (*0.94–0.98*)***	0.99* (*0.97–1.0*)	1.00 (*0.97-1.03*)

Male	1.0	1.0	1.0
Female	0.97 (0.79-1. 18)	1.27 (1.12 – 1.42)***	1.29 (0.99-1.65)
Age >75 years	1.0	1.0	1.0
Age 65–74 years	1.5 (1.19-1.89)**	0.74 (0.65 - 0.84)***	1.13 (0.84-1.5)
Age 50–64 years	1.49 (1.15-1.93)***	0.64 (0.55 - 0.74)***	1.0 (0.72-1.41)
Distal subsite	1.0		
Proximal subsite	1.71 (1.36-2.13)***		
Unknown site	0.95 (0.73-1.25)		
Non-MPA		1.0	1.0
MPA		1.79 (1.42-2.26)***	2.48 (1.73-3.66)***
Type not ascertained		1.54 (1.3-1.8)***	3.44 (1.19-9.86)**

* *P*<0.05,

** *P*<0.01,

*** *P*<0.001.MPA=mucin-producing adenocarcinoma.

† effect of one unit change in deprivation score.

). MPA tumour type was significantly more likely in younger patients and in patients with proximal subsite, while there was no effect of sex.

Having adjusted for sex, age group and subtype, the likelihood of proximal colon cancer showed little variation between SE groups and no clear gradient (Table 2 –model 2). The test for trend, however, showed a marginally significant lower risk of proximal sub site with increasing levels of deprivation. This was largely due to the effect of the most-deprived group, accounting for 40% of all cases, suggesting a threshold rather than a continuous effect of increasing deprivation in the probability of proximal tumours. Proximal subsite was significantly more likely among women and patients with MPA or unknown subtype, and significantly less likely in the 50–74 years age group.

Owing to a strong period effect in grade completeness, only cases post 1993 were included in model 3. Having adjusted for sex, age group and subtype, the likelihood of poor grade showed no clear association with SE status (Table 2 – model 3). Women had a marginally non-significant excess risk of poor-grade tumours. Patients with MPA and unknown subtype tumours were significantly more likely to have poorly differentiated tumours. There was no consistent effect of age.

## DISCUSSION

The results show that more deprived colon cancer patients do not have an excess risk of suffering from colon tumours of MPA subtype, proximal subsite and poor grade. Therefore, findings do not support the hypothesis that more deprived colon cancer patients are more likely to have tumours with characteristics associated with poorer prognosis. Indeed, the results show that least-deprived (affluent) patients are more likely to have tumours of MPA subtype and proximal subsite.

It is possible that there has been a degree of under-ascertainment of MPA subtype in the study dataset −6.4% compared with about 13% reported in US studies (Mayberry *et al*, 1995; Chen *et al*, 1997), although it is unclear whether this difference represents under-diagnosis of MPA subtype in the UK or overdiagnosis in the US. For MPA-type underdiagnosis to bias the results, the quality of histopathological diagnosis would have had to differ by deprivation status, which is unlikely. Pathologist inter- and intraobserver variation in the classification of MPA type could not be controlled for, but this would be expected to weaken rather than exaggerate the observed association with SE status. As previously reported (Mayberry *et al*, 1995), there is a higher risk of MPA tumour subtype in younger patients. There was a previously unreported, excess risk of MPA subtype in patients with proximal subsites tumours and in women.

The marginally significant lower risk of proximal subsite with increasing levels of deprivation contrasts with a French study, showing inverse findings (Faivre *et al*, 1989). The findings, however, are more consistent with studies showing excess risk of rectal cancer in deprived patients (Ferraroni M *et al*, 1989; Kee *et al*, 1996), and with suggestions that colon subsites should ideally be treated differently in analytical epidemiological research. There was an excess likelihood of proximal colon subsite in women, as observed previously (Vobecky *et al*, 1984; Jass, 1991; Levi *et al*, 1991; Chen *et al*, 1997). The lower likelihood of proximal tumours in young age contrasts with previous research showing an inconsistent effect of age (Jass, 1991).

There was a lack of clear SE gradient in the risk of poor-grade colon cancer. MPA-type tumours are more likely to be poorly differentiated, which could partially explain the poor prognosis associated with this subtype. There was a previously unreported and significant excess risk of poor-grade tumours in women.

In common with previous UK research in this field, SES was measured ecologically. The theoretical possibility of a true effect of SES on colon cancer tumour factors not having been detected due to misclassification error is acknowledged. Similarly, examined tumour factors might have been unsuitable to explore the study hypothesis and other tumour characteristics, such as vascular invasion and newer biochemical and genomic markers might have been more suitable. However, most ‘newer’ markers are correlated with ‘conventional’ tumour factors such as the ones examined here, making it likely that their SE distributions would also be similar. It was not possible to examine the influence of tumour stage; however, stage *per se* is a poor indicator of intrinsic disease severity, as it can be influenced by patient and healthcare factors (e.g. timeliness of self-referral and investigation). Based on present evidence therefore, it is unlikely that tumour factors could account for observed SE differences in colon cancer survival. Future research should concentrate on the potential influence of differences in healthcare and patient factors.
